# Social–ecological landscape patterns predict woody encroachment from native tree plantings in a temperate grassland

**DOI:** 10.1002/ece3.4340

**Published:** 2018-09-05

**Authors:** Victoria M. Donovan, Jessica L. Burnett, Christine H. Bielski, Hannah E. Birgé, Rebecca Bevans, Dirac Twidwell, Craig R. Allen

**Affiliations:** ^1^ Department of Agronomy and Horticulture University of Nebraska Lincoln Nebraska; ^2^ Nebraska Cooperative Fish and Wildlife Research Unit School of Natural Resources University of Nebraska Lincoln Nebraska; ^3^ U.S. Geological Survey – Nebraska Cooperative Fish and Wildlife Research Unit School of Natural Resources University of Nebraska Lincoln Nebraska

**Keywords:** afforestation, agroforestry, *Juniperus virginiana*, tree planting, windbreak, woody encroachment

## Abstract

Afforestation is often viewed as the purposeful planting of trees in historically nonforested grasslands, but an unintended consequence is woody encroachment, which should be considered part of the afforestation process. In North America's temperate grassland biome, Eastern redcedar (*Juniperus virginiana* L.) is a native species used in tree plantings that aggressively invades in the absence of controlling processes. Cedar is a well‐studied woody encroacher, but little is known about the degree to which cedar windbreaks, which are advocated for in agroforestry programs, are contributing to woody encroachment, what factors are associated with cedar spread from windbreaks, nor where encroachment from windbreaks is occurring in contemporary social–ecological landscapes. We used remotely sensed imagery to identify the presence and pattern of woody encroachment from windbreaks in the Nebraska Sandhills. We used multimodel inference to compare three classes of models representing three hypotheses about factors that could influence cedar spread: (a) windbreak models based on windbreak structure and design elements; (b) abiotic models focused on local environmental conditions; and (c) landscape models characterizing coupled human‐natural features within the broader matrix. Woody encroachment was evident for 23% of sampled windbreaks in the Nebraska Sandhills. Of our candidate models, our inclusive landscape model carried 92% of the model weight. This model indicated that encroachment from windbreaks was more likely near roadways and less likely near farmsteads, other cedar plantings, and waterbodies, highlighting strong social ties to the distribution of woody encroachment from tree plantings across contemporary landscapes. Our model findings indicate where additional investments into cedar control can be prioritized to prevent cedar spread from windbreaks. This approach can serve as a model in other temperate regions to identify where woody encroachment resulting from temperate agroforestry programs is emerging.

## 1 INTRODUCTION

Afforestation (the conversion of historically nonforested lands to forests) is leading to the loss of grassland ecosystems across multiple continents (Briggs et al., 2005; Fensham, Fairfax, & Archer, 2005; Roques, O'Connor, & Watkinson, 2001). Afforestation is often viewed as the purposeful planting of trees in grasslands; however, woody encroachment is an unintended consequence of planting native trees in temperate grassland regions and should be considered as part of the afforestation process (Veldman, Overbeck, Negreiros, Mahy, Le Stradic et al., 2015). Agroforestry programs have used potential social–ecological benefits to justify the use of native tree species in afforestation programs and assumed those species would not incur the types of unintended impacts consistent with the planting, and subsequent invasion, of exotic tree species (Ganguli, Engle, Mayer, & Fuhlendorf, 2008; Montagnini, Cusack, Petit, & Kanninen, 2004; Richardson, 1998). It is now clear, however, that there are unintended consequences when using native trees in temperate afforestation programs (e.g., Ratajczak, Nippert, & Collins, 2012; Steinauer & Bragg, 1987; Twidwell et al., 2013). Increases in woody cover drive declines in native grassland species richness and diversity (Ratajczak et al., 2012; Sirami, Seymour, Midgley, & Barnard, 2009) and lead to the loss of a suite of ecosystem services (Twidwell et al., 2013). Yet little is known about the processes in social–ecological landscapes that shape encroachment from native tree plantings into the wider landscape matrix.

Afforestation of the world's grasslands and savannahs has been backed by national and international governing bodies for the last century (e.g., Ganguli et al., 2008; Gardner, 2009; Veldman, Overbeck, Negreiros, Mahy, Stradic et al., 2015). Historically, disturbance patterns like frequent fire limited the spread of trees into many grassland regions (Bond, Woodward, & Midgley, 2005). A combination of fire suppression, the elimination of megafaunal herbivores, and tree plantings have led to rapid tree expansion into many grassland regions across the globe (Parr, Lehmann, Bond, Hoffmann, & Andersen, 2014; Veldman, Overbeck, Negreiros, Mahy, Stradic et al., 2015). Despite scientific evidence that suggests the drastic costs of afforestation in grassland systems (Berthrong, Jobbágy, & Jackson, 2009; Jackson et al., 2005), tree planting remains a common practice. In many instances, this results in a double‐think mentality that creates contradictory policies for the promotion and control of woody species (e.g., Roberts, Uden, Allen, & Twidwell, 2018). Determining patterns of spread from tree plantings can establish a better understanding of the contribution of tree planting to afforestation and allow for improved management of woody encroachment from tree plantings.

**Figure 1:**
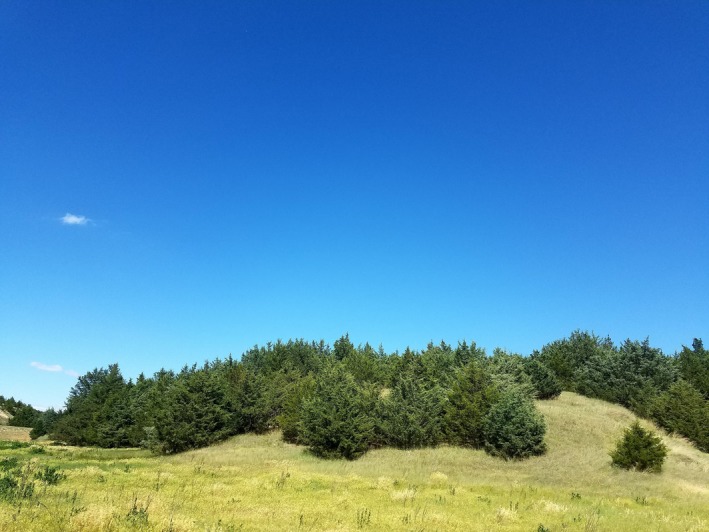
Eastern redcedar (*Juniperus virginiana* L.) invading a grassland. Photograph courtesy of Christine H. Bielski

In North America's grassland biome, the planting of eastern redcedar (*Juniperus virginiana* L.; hereafter cedar; Figure 1) has been a government‐backed program for more than 100 years (Ganguli et al., 2008; Gardner, 2009). In 2001 alone, more than 1.8 million cedar trees were distributed for planting in the Great Plains (Ganguli et al., 2008). Cedars are most often planted as windbreaks, or rows of trees, used to provide shelter around buildings, reduce wind‐facilitated cropland erosion, and to provide shelter for livestock from extreme weather (Ganguli et al., 2008). Cedar acts as a rapid colonizer of grassland areas in the absence of recurrent disturbances (Engle, Coppedge, & Fuhlendorf, 2008; Twidwell et al., 2013). Fire suppression in association with government and citizen initiatives to afforest the Great Plains following European settlement has led to cedar expansion throughout the biome (Engle et al., 2008). Cedar spread from windbreaks has been documented for decades (Graf, 1965; Smith, 1986). Rapid increases in cedar cover initiate swift and profound changes in ecological structure and functioning, including altered aboveground biomass allocation, nutrient cycling, ecosystem productivity, soil chemistry, and water table (McKinley, Norris, Blair, & Johnson, 2008; Mellor et al., 2013; Wilcox & Thurow, 2006). The social outcomes of such changes include a loss of grazing lands, decreased wildfire suppression potential, changes in groundwater recharge, and a loss of grassland biodiversity (Twidwell et al., 2013). Despite scientific evidence implicating woody encroachment as a leading driver of change in the Great Plains (Briggs, Hoch, & Johnson, 2002; Twidwell et al., 2013), U.S. state and federal agencies (e.g., National Wildlife Organization; Natural Resources Conservation Service Environmental Quality Incentives Program) continue to support plantings and renovation of cedar windbreaks.

Tree plantings serve as a propagule source for woody encroachment, but they are embedded in a larger landscape matrix where multiple social and ecological drivers determine the grassland–woodland interface. Invasion biologists agree that adequate propagule pressure is necessary for species establishment in a region (Lockwood, Cassey, & Blackburn, 2005; Simberloff, 2009). Demographic and structural features of plantings can influence the amount of propagule pressure in the surrounding landscape. Abiotic factors, such as precipitation gradients, have been linked to patterns in the coexistence of woody and grass vegetation globally (e.g., Sankaran et al., 2005). In many regions in the Great Plains, it is assumed that abiotic condition prevents the spread of cedar from windbreaks. Moreover, woody encroachment is being more strongly tied to social influences (Engle et al., 2008). Hoch and Briggs (1999) demonstrate a strong relationship between human populations, anthropogenic features, and woody encroachment. Berg et al. (2015) emphasized the importance of human distributions on woody encroachment, revealing that patterns of woody encroachment cannot be explained solely by ecological drivers. Thus, windbreak proximity to coupled social–ecological landscape characteristics can influence woody encroachment from plantings.

The afforestation debate is one of the biggest challenges to grassland conservation in the Nebraska Sandhills, one of the largest contiguous grassland in North America. Consistent with many agroforestry programs, it is assumed that cedar trees are either (a) not spreading from windbreaks or (b) control measures are in place to halt spread. Our objectives were to use remotely sensed imagery to (1) determine whether encroachment is occurring from windbreaks in the Nebraska Sandhills; (2) disentangle which of the three hypotheses describing woody encroachment from windbreaks is best able to predict the presence of cedar spread through model selection; and (3) identify the relationship between cedar spread and each predicting variable in our top model.

## 2 MATERIALS AND METHODS

### 2.1 Study area

The Nebraska Sandhills in the north‐central Great Plains of the United States is one of the largest contiguous grasslands in North America, encompassing more than 50,000 km^2^. It is the largest grass‐stabilized sand dune region in the western hemisphere (Bleed & Flowerday, 1990), and consists of dunal uplands, dry valley floors, subirrigated meadows, small lakes, and wetlands (Gosselin, Sridhar, Harvey, & Goeke, 2006; Rundquist, 1983). Rangelands dominate the land use of this region, meaning the majority of grasslands are grazed by cattle (Volesky, Schacht, Reece, & Vaughn, 2005). Wildfires are rare, with only ~1% of the region being burned by 14 wildfires >400 ha in the last decade (Donovan, Wonkka, & Twidwell, 2017). Likewise, prescribed fire on private rangelands is minimal (Ortmann, Stubbendieck, & Mitchell, 2007).

Cedar windbreaks have been planted in the Nebraska Sandhills for ~150 years and are subsidized by state and federal governments (Ganguli et al., 2008). The Sandhills is one of the only ecoregions left in the central Great Plains that has large portions of grasslands not yet converted to cedar woodland, allowing us to assess early patterns of encroachment when sources of cedar spread are localized (Ortmann et al., 2007). Windbreaks are considered the primary source of localized infestations of cedar in this region (Ortmann et al., 2007). Dispersal of cedar beyond the windbreak canopy is driven primarily by wildlife (Holthuijzen & Sharik, 1985; Horncastle, Hellgren, Mayer, Engle, & Leslie, 2004).

### 2.2 Windbreak selection

**Figure 2:**
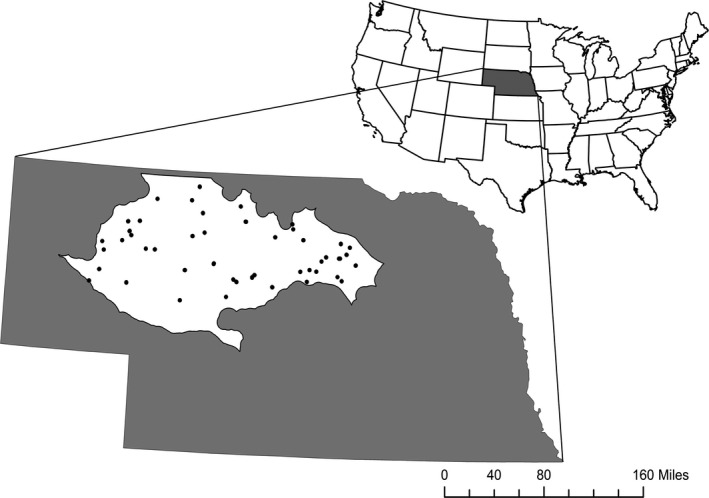
Map of the 10‐km interior‐buffered conterminous Nebraska Sandhills ecoregion (area in white) and the sampling locations of eastern redcedar (*Juniperus virginiana* L.) windbreaks within this region (black dots)

The Nebraska Sandhills is 98% private land, and therefore, monitoring data on woody encroachment is extremely limited. Thus, we identified individual windbreaks using high‐resolution remotely sensing imagery from 1993 to 2013 compiled by Google Earth (v. 7.1.8.3036, http://www.google.com/earth). Remotely sensed imagery provides a record for analyzing woody plant abundance where no other monitoring efforts exist, and has been used effectively in similar studies focused on shrubby encroachment in United States grasslands (Briggs et al., 2002; Laliberte et al., 2004; McKinley et al., 2008). To select individual windbreaks as sampling units, we generated 50 random points that were distributed within a 10‐km interior‐buffered region of the conterminous Sandhills ecoregion (Omernik, 1987; Figure 2). We then identified the nearest windbreak to each point that was >500 m from a riparian area (where cedar tends to be prolific), and >100 m from the nearest cedar windbreak or stand, to remove potential confounding sources of propagules that could influence our measures of encroachment from selected windbreaks. Candidate windbreaks were also required to have at least one “side” free from (>100 m) visible rowcrop, plowed fields, gray infrastructure, or roads. All windbreaks were selected using the most recent (2013) imagery.

### 2.3 Measuring woody encroachment

**Figure 3:**
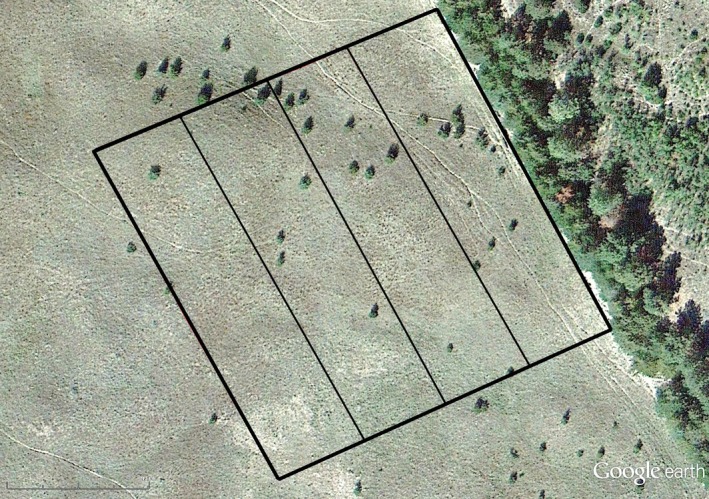
An example of a 100 m by 100 m belt transect overlain on a 2013 Google Earth v. 7.1.8.3036 remotely sensed image (41.86°, −100.48°) used to determine the presence of eastern redcedar (*Juniperus virginiana* L.) spread from a windbreak. The sample area was divided into four sections to assist with searching for cedar

We randomly selected the direction from which we measured woody encroachment from each windbreak; however, if a candidate windbreak was adjacent to man‐made structures or agriculture (e.g., rowcrop, roads) where cedar is unable to establish on one side, the opposite side was used. All windbreaks had evidence of grazing in the surrounding pastures. We delineated a 100 m belt transect extending from the center of each windbreak and spanning 100 m in width to create a 100 m by 100 m rectangle (Figure 3) in which we documented the presence or absence of cedar trees at a single time‐step using 2013 colored imagery. Although cedar propagules can spread over vast distances, we felt that limiting our assessment to a small belt transect bordering the windbreak would give the highest probability that the woody encroachment we recorded resulted from the windbreak of interest.

### 2.4 Model development

We developed three classes of models representing three scales of factors that could influence cedar spread: windbreak (patch) models, local abiotic models, and landscape models (Table 1). This allowed us to contrast three primary hypotheses about cedar proliferation from windbreaks: (a) cedar spread from windbreaks is most strongly associated with windbreak characteristics linked to propagule pressure; (b) cedar spread from windbreaks is most strongly associated with abiotic conditions such as precipitation patterns and soils; or (c) cedar spread from windbreaks is most strongly associated with social–ecological landscape patterns.

**Table 1:** Candidate models created to model the probability of eastern redcedar (*Juniperus virginiana* L.) spread at three different scales: windbreak (patch) models, local abiotic models, and landscape models

Model number	Model predictors	Variable type
Windbreak models		
1	Density + age + area	
2	Age	Binary
3	Density	Binary
4	Area	Continuous
Abiotic models		
5	Soil + longitude + latitude + slope	
6	Soil	Continuous
7	Longitude	Continuous
8	Latitude	Continuous
9	Slope	Continuous
Landscape models		
10	Distance to cedar + distance to water body + distance to farmstead + distance to road	
11	Distance to cedar	Continuous
12	Distance to water body	Continuous
13	Distance to farmstead	Continuous
14	Distance to road	Continuous

Windbreak models represented patch‐scale characteristics of windbreaks that could influence propagule pressure in the surrounding landscape, including *age*,* density*,* area*, and *width* (Table 1). We classified *age* as a binary variable (<30 years or >30 years), with a 30‐year cutoff representing the approximate age at which maximum seed production begins (Smith, 1986). We considered windbreaks that appeared fully matured in 1993 imagery to be >30 years old, and those that showed continual growth from 1993 to 2013 to be <30 years old. We estimated *density* as the amount of canopy cover within an entire windbreak in the year 2013 (<75% or >75% cover) following the methods of the USDA Forest Service (2012). We calculated windbreak *area* and *width* in ArcMap (v. 10.3) using the polygon and ruler tools, respectively.

Abiotic models consisted of *soil*,* latitude*,* longitude,* and *slope* (Table 1). We classified *soil* as the percent of sand in the surface layer at the central point of the windbreak using SSURGO's Soil Data Viewer (version 6.2; Soil Survey Staff, NRCS). Slope was measured in Google Earth by subtracting changes in elevation from the edge of the windbreak to the end of our 100 m belt transect. *Latitude* and *longitude* were recorded at the center of each windbreak using ArcGIS software to represent gradients in local climatic conditions such as precipitation and temperature.

Landscape models consisted of features within the grassland matrix that might influence spread, including *distance to cedar*,* distance to water body* (a temporary pond, a lake or a river), *distance to road*, and *distance to farmstead* (Table 1). All measures were taken as the distance (m) from the center of each windbreak to the closest edge of the respective feature. Although all windbreaks fell within the Sandhills, three fell well outside of our 10 km interior‐buffered region. Thus, we chose to remove these windbreaks from our modeling data set to reduce the chance of edge effects (Figure 2).

We tested for multicollinearity among all predictor variables. Two variables were strongly correlated (*r* > 0.60): windbreak *width* and *area*. We removed *width* from all analyses. Our final candidate model set consisted of 14 generalized linear (logit link) models (Table 1).

Prior to model selection, we tested each inclusive model (*N* = 3) and the intercept‐only model for spatial autocorrelation of our binary response variable (encroachment or no encroachment of cedar about the windbreak) by inspecting the semivariance over increasing distances among the spatial coordinates of cedar windbreaks. We found no evidence of spatial autocorrelation. We also assessed the goodness of fit for each inclusive model (*N* = 3) using the Hosmer and Lemeshow goodness‐of‐fit test (R package *Resource Selection*; Lele, Keim, & Solymos, 2016). Goodness‐of‐fit tests indicated no significant deviance from the models and the observed data (windbreak inclusive model (model 1): $\chi_{8}^{2}=6.69$, *p* = 0.57; biophysical and propagule escape inclusive model (model 5): $\chi_{8}^{2}$ = 2.96, *p* = 0.94; coupled social–ecological effects inclusive model (model 9): $\chi_{8}^{2}$ = 4.28, *p* = 0.50), suggesting that each of our global models were a good fit to our data.

### 2.5 Model selection

We used model selection techniques (Burnham & Anderson, 2002) to identify which model(s) best describe the spread of cedar from windbreaks in our study area. We used Akaike's Information Criterion corrected for small sample sizes (Akaike, 1973) to determine the best model(s) (package *AICcmodavg;* Mazerolle, 2016). We considered models within two AICc values (ΔAICc ≤ 2) of the top model (ΔAICc = 0) to be the “best model(s).” All statistical analyses were conducted in r statistical software (v. 3.3).

## 3 RESULTS

### 3.1 Objective 1

Cedar is spreading from tree plantings in the Nebraska Sandhills. Woody encroachment occurred at 23% of the windbreaks that we assessed (*N* = 47). The density of cedar spread within our 100 m belt transect was variable, ranging from 1 to 342 trees per ha. Windbreaks were an average length of 334 m ± 24 *SE* and an average area of 9,690 m^2^ ± 1,512 *SE*.

### 3.2 Objective 2

Of our three hypotheses, the inclusive landscape model (model 9) representing windbreak proximity to social–ecological landscape features (distance to cedar + distance to water body + distance to farmstead + distance to road) best predicted the presence of cedar spread from windbreaks. This model carried 92% of the AICc weight among our candidate model set (Table 2; McFadden *R* ^2^ = 0.41), indicating that there was a 92% probability that this model was the best model from our model set to predict the presence of cedar spread from windbreaks. The next highest ranking model (Model 13; Distance to farmsteads) was in this same model class and carried 4% of AICc weight.

**Table 2:** Relative support for candidate models explaining variation in the presence of eastern redcedar (*Juniperus virginiana* L.) encroachment from windbreaks in our sampling area of the Nebraska Sandhills ecoregion

Model	Model description	*K* a	LL^b^	AICc^c^	∆AICc^d^	*w* (%)^e^
10	Distance to cedar + distance to water body + distance to farmstead + distance to road	5	−15.01	41.48	0.00	92
13	Distance to farmstead	2	−21.72	47.71	6.23	4
12	Distance to water body	2	−23.18	50.62	9.15	1
5	Soil + longitude + latitude+ slope	5	−19.79	51.04	9.56	1
7	Longitude	2	−23.62	51.51	10.03	1
8	Latitude	2	−23.96	52.20	10.72	0
15	1	1	−25.57	53.24	11.76	0
11	Distance to cedar	2	−24.85	53.98	12.50	0
4	Area	2	−25.05	54.37	12.89	0
9	Slope	2	−25.20	54.68	13.20	0
2	Age	2	−25.40	55.07	13.60	0
14	Distance to road	2	−25.56	55.40	13.92	0
3	Density	2	−25.56	55.40	13.92	0
6	Soil	2	−25.56	55.40	13.92	0
1	Density + age + area	4	−24.99	58.94	17.46	0

^a^Number of estimated parameters included in the model. ^b^Logarithm of maximum likelihood for each model. ^c^Akaike information criterion adjusted for small sample size. ^d^Difference in Akaike's information criterion adjusted for small sample size from the best model. ^e^Akaike weight for each model; rounded to the nearest whole number.

The likelihood of other models was negligible. The inclusive abiotic environment model was the highest ranked model from this model class and had only 1% of model weight, indicating low support. There was zero probability that any of the windbreak models, representing potential variability in propagule pressure, explained the presence of cedar spread from windbreaks we assessed in the Nebraska Sandhills.

### 3.3 Objective 3

**Figure 4:**
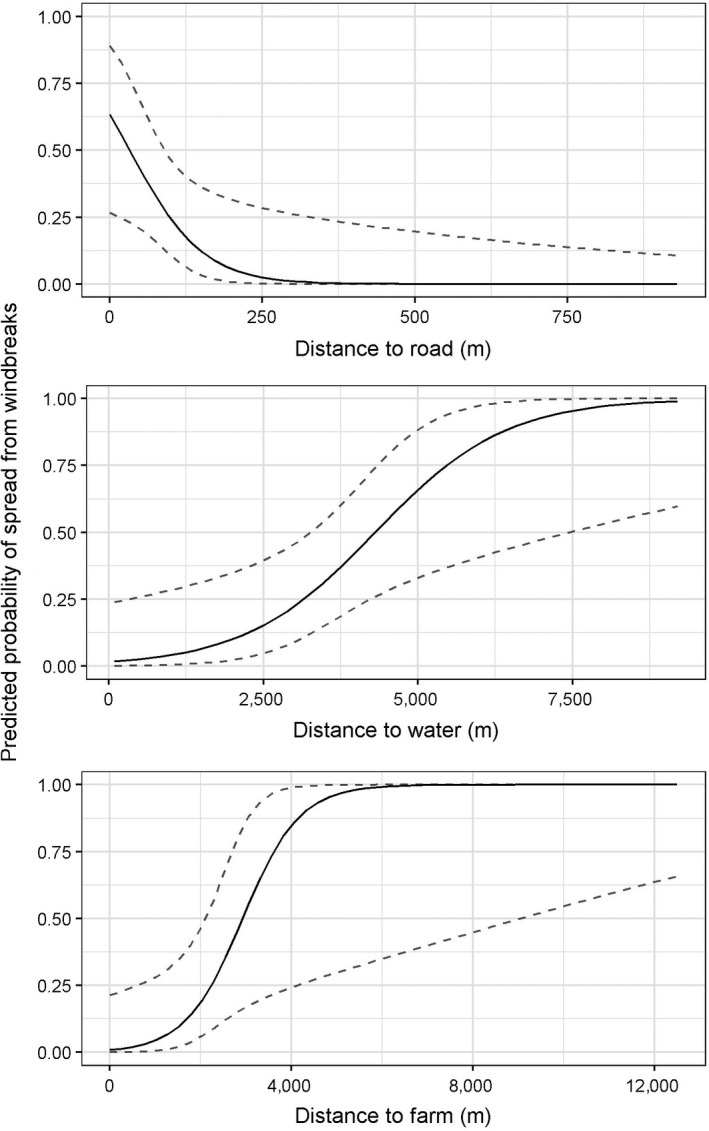
Predicted probabilities (95% confidence intervals) of eastern redcedar (*Juniperus virginiana* L.) encroachment from windbreaks with respect to the distance of a windbreak from (a) dirt or paved road, (b) a waterbody, and (c) a farmstead

The likelihood of cedar spread is negatively related to the distance to roads (*β* = −0.017, *SE* = 7.77e‐3; Figure 4), meaning that cedar spread from a windbreak is more likely to occur when the windbreak is closer to a road. There is a positive relationship between cedar spread and the distance to farmsteads (*β* = 0.0016, *SE* = 7.22e‐4; Figure 4), indicating that cedar windbreaks are less likely to have spread present if they are closer to a farmstead. There is a similar relationship between windbreaks and distance to waterbodies (*β* = 0.00095, *SE* = 3.83e‐4; Figure 4). Likewise, there is a greater likelihood of the presence of cedar spread at windbreaks that are farther from other cedar patches (*β* = 0.0026, *SE* = 3.83e‐4).

## 4 DISCUSSION

An explicit focus on temperate agroforestry programs can provide the data necessary to move the academic criticism of temperate afforestation practices toward a more data‐driven assessment. We provide evidence of the contribution of woody encroachment from tree plantings to grassland afforestation. Temperate agroforestry operates under the assumption that using native tree plantings will halt spread into the surrounding environment and will therefore have less detrimental impacts compared to exotic species, or that controls are in place to prevent woody encroachment (Ganguli et al., 2008; Montagnini et al., 2004; Soutar & Peterken, 1989). This assumption is not consistent with the patterns of woody encroachment observed in this study, and a body of research already exists to show woody encroachment has major trade‐offs in temperate grasslands (Twidwell et al., 2013). Native invaders have not been given the same attention as invasive alien species, even though their impacts are structurally and functionally similar (Nackley, West, Skowno, & Bond, 2017). Conflicting messages between the science and management of native invaders can lead to double‐think policies and the simultaneous promotion and control of species (Roberts et al., 2018).

The proximity of tree plantings to social–ecological landscape features described cedar spread from windbreaks in the contemporary Nebraska Sandhills decidedly better than windbreak characteristics associated with propagule pressure and abiotic characteristics of the surrounding landscape. Abiotic gradients, like precipitation gradients, have been strongly tied to woody plant prevalence globally (e.g., Sankaran et al., 2005). Our results suggest that abiotic environment does not limit the spread of woody vegetation from tree plantings in the Nebraska Sandhills, indicating that the entire sandhills region is vulnerable to woody encroachment. Likewise, windbreak characteristics that influence propagule pressure in the surrounding landscape were negligible in describing the presence of cedar spread. A single cedar tree can produce up to 4.4 million seeds in a given year (Stoeckler & Slabaugh, 1965). Management attempts to manipulate windbreak density or structure may not alter the probability of woody encroachment. Instead, our results suggest that it is likely the propensity of landowners and managers to manage certain areas more intensely than others that determines the presence of cedar spread across the landscape.

We found that cedar spread was less likely surrounding windbreaks near farmsteads, waterbodies, and other cedar patches, while encroachment was more likely surrounding windbreaks near roadways. Landowners and managers have a strong influence on the magnitude and direction of woody encroachment (Schmidt & Leatherberry, 1995), and control efforts such as herbicide application and manual removal tend to be implemented in areas near human development (Coppedge, Engle, & Fuhlendorf, 2007). Local patterns of disturbance (e.g., fire and grazing) can prevent woody establishment (Archer et al., 2017). Indeed, 87% of windbreaks did not have detectable woody encroachment, indicating that controlling processes are likely effective in these areas. However, recurrent management may be absent, or at least insufficient, near and along roadways, thereby providing new opportunities for propagules to escape. Woody plants must escape injury from disturbances in order to pass from the seedling to adult stage (Bond & Midgley, 2000). Management varies based on differences in landowner motivations and personal histories (Berg et al., 2015; VanWey, Ostrom, & Meretsky, 2005). Human infrastructure and increasing fragmentation driven by exurban and urban sprawl may unknowingly promote increased cedar spread from windbreaks by providing refuges for cedar based on the propensity to manage in certain areas and not others (Coppedge, Engle, Fuhlendorf, Masters, & Gregory, 2001a,b; Coppedge et al., 2007).

It is important to consider that the processes responsible for establishment and spread differ (Allen et al., 2013). Our study investigated cedar spread, rather than establishment. Because of the limitations associated with spatial resolution in remotely sensed imagery, we modeled patterns in encroaching cedar trees that were large enough to detect using this data source. Thus, spread associated with smaller trees, particularly those at or below grass height, is not represented in our analysis. It is also important to consider that factors affecting woody encroachment will differ depending on the scale of assessment (e.g., factors influencing the encroachment of an entire watershed likely differ from the processes affecting encroachment in a pasture). The scales of our assessment should be considered when applying our results. Finally, interactions among people and nature differ across global temperate grassland regions, meaning that the patterns in woody encroachment observed in the Sandhills should be expected to differ from other temperate grassland regions.

Moving toward a more scientifically based view of afforestation is necessary to better balance trade‐offs of afforestation of the world's grassland ecosystems. We demonstrate that one of the fundamental assumptions of native species use in agroforestry programs, that native species would not incur the types of unintended impacts consistent with the planting, and that subsequent invasion, of exotic tree species, is incorrect (Ganguli et al., 2008; Montagnini et al., 2004; Richardson, 1998). Even when control efforts are implemented in a grassland region, native species can spread from tree plantings to further contribute to the loss of grassland ecosystem services and biodiversity (Twidwell et al., 2013). Ecological misconceptions about global grassland and forested ecosystems need to be remedied to halt the continued loss of grassland ecosystems across the globe (Veldman Overbeck, Negreiros, Mahy, Le Stradic et al., 2015).

## CONFLICT OF INTEREST

No competing interests to declare.

## AUTHOR CONTRIBUTIONS

CA, VD, JB, HB, CB, and RB conceived the ideas and designed the methodology. VD, CB, JB, and RB collected the data. JB analyzed the data. VD and DT led the writing of the manuscript. All authors contributed critically to drafts and gave final approval for publication.

## DATA ACCESSIBILITY

Data from this study will be deposited in Dryad Digital Repository following manuscript acceptance.
